# Anxiety, depression and post-traumatic stress disorder in patients on hemodialysis in the setting of the pandemic, inflation, and the Beirut blast: a cross-sectional study

**DOI:** 10.1186/s12888-023-04798-6

**Published:** 2023-04-22

**Authors:** Rita Khoury, Ziad Ghantous, Roy Ibrahim, Elias Ghossoub, Patille Madaghjian, Elie Karam, Georges Karam, Najat Fares, Sabine Karam

**Affiliations:** 1 Department of Psychiatry, American University of Beirut, PO Box: 11-0236, Beirut, 1107 Riad El Solh Lebanon; 2 Department of Psychiatry, Washington University in St. Louis, Clayton, Missouri United States; 3 Department of Psychiatry and Clinical Psychology, Saint Georges Hospital University Medical center, Beirut, Lebanon; 4 Department of Nutrition, Saint Georges Hospital University Medical center, Beirut, Lebanon; 5 Department of Internal Medicine, Division of Nephrology, Saint Georges Hospital University Medical center, Beirut, Lebanon; 6 Division of Nephrology and Hypertension, University of Minnesota, Minneapolis, United States

**Keywords:** Anxiety, Depression, Post-traumatic stress disorder, Suicidality, Cognitive impairment, Hemodialysis, Explosion, Trauma, Lebanon

## Abstract

**Background:**

In 2020, Lebanon has witnessed its worst economic crisis, exacerbated by the COVID-19 pandemic and a massive explosion of its capital. Amidst these stressors, this study aims at assessing the prevalence of depression, anxiety, suicidality, post-traumatic stress disorder (PTSD) and cognitive impairment in patients undergoing hemodialysis in an academic hospital destroyed by the explosion.

**Methods:**

This cross-sectional study conducted 6 months after the blast included adults on hemodialysis, with no previous diagnoses of dementia or intellectual disability. It explores prevalence rates of psychiatric disorders, in addition to other medical and psychosocial variables such as frailty, malnutrition, sarcopenia, quality of life and religiosity.

**Results:**

Forty two patients (mean age 66.1; SD: 11.2 years) undergoing hemodialysis for 6.12 years (SD:7.22 years) were included. Anxiety and depression rates reached 54.8% and 57.1% using cut-offs of 6 and 7 respectively on the Hospital Anxiety and Depression rating Scale. 9.5% of the patients reported being in the hospital at the time of the blast and 7.1% reported being injured. 33.3% screened positively for PTSD using a cut-off of 23 on the PCL-5. 26.2% had passive death wishes and 7.1% had suicide plans, however no one had attempted it. 23.8% were found cognitively impaired as shown by the Mini-Cog (<3). Around two-third of participants were moderately to severely malnourished per the GLIM criteria. One third suffered from frailty, according to the FRAIL screening tool. Around 60% suffered from sarcopenia, based on handgrip strength measures. These findings contrast with “acceptable to good” quality of life subjectively reported by participants on the Short Form 36 (SF-36) Health Survey. While one-third of participants participated in organizational religious activities, 88% reported significant subjective meaning of religion in their heart.

**Conclusions:**

Rates of depression, anxiety, PTSD, suicidality, and cognitive impairment were found to be alarming in the setting of an urban dialysis unit following a major explosion. Psychiatric disorders were found to be compounded with increased prevalence of malnutrition, frailty, and sarcopenia. These findings urge healthcare providers to implement early diagnostic and intervention strategies to improve both mental and physical wellbeing of this vulnerable population, in similar settings.

## Introduction

On August 4, 2020, Beirut the capital city of Lebanon, a small Mediterranean lower middle-income country, witnessed a major blast in its port that led to more than 200 dead and 7000 injured [1]. The destruction from the explosion left around 300 000 residents with devastated dwellings [2]. In addition, several hospitals were partially or completely destroyed. Saint Georges Hospital University Medical Center (SGHUMC), a major tertiary-care center that faces the port directly, was subject to severe damage and became non-functional for around a month after the explosion. A considerable number of patients and staff members present in the hospital at the time of the explosion were injured or killed and subject to physical and psychological trauma, including patients who were undergoing their hemodialysis shift at the time of the blast.

Patients with hemodialysis have a high risk of developing comorbid psychiatric illnesses such as depression, anxiety, and suicidal ideations [3]. For example, the prevalence of anxiety disorders in patients on hemodialysis ranges between 12-52% compared to 8% in primary care settings [4]. The prevalence of depression is even higher, reaching 85% in certain sample populations [5]. In addition, the lifetime prevalence of post-traumatic stress disorder (PTSD) independent from trauma type is 17% in hemodialysis patients and drops down to 10% when hemodialysis itself is perceived as the traumatic event. PTSD is associated with depression, anxiety, and poor quality of life [6]. Depression and anxiety in patients undergoing hemodialysis also affect their quality of life and adherence to treatment [7].

In addition to the physical and psychological trauma associated with the Beirut explosion, COVID-19 exposure/infection alongside the accompanying social isolation/quarantine can be perceived as potential traumatic events, due to the life-threatening aspect of the pandemic at that time. In fact, a survey done on 116 hemodialysis patients during the Middle East Respiratory Syndrome (MERS) outbreak in South Korea in 2015 revealed post-traumatic stress symptoms in those who were isolated for a certain period of time because of the infection (around 18% of the sample) [8].

Furthermore, the country has been going through its worst humanitarian and economic crisis, with inflation rates reaching 281% between June 2019 and 2021, and its national currency losing more than 90% of its value since October 2019, all of which had severely impacted the health care sector, according to a 2022 report by the Human Rights Watch [9]. Adding to that, the blast’s economic burden exceeded 6.7 billion US dollars, which added to the magnitude of the economic and healthcare crisis [10].

One previous study conducted in 2012 at another Lebanese university hospital (Hotel Dieu de France Hospital) in Beirut using the Hospital Anxiety and Depression Scale-HADS found depression and anxiety rates among 51 patients undergoing hemodialysis to be 50 and 45% respectively [11]. In another study done in 2017 at the American University of Beirut, around 40% of patients on hemodialysis were found to suffer from depression or anxiety. One quarter of patients suffered from both conditions [12].

In the setting of the explosion and the COVID-19 pandemic, our current study aims to explore the prevalence of depression, anxiety, suicidality, and post-traumatic stress symptoms/disorder in end stage kidney disease patients undergoing their dialysis at SGHUMC. Patients were temporarily displaced to other hospitals to continue their treatment for 5 weeks, before returning to SGHUMC.

## Methodology

### Study design and conduct

This is a cross-sectional study that was conducted between March and April 2021 (6 months after the blast) among adult patients, undergoing hemodialysis on a chronic basis at the outpatient unit of SGHUMC. Excluded were patients with a previously established diagnosis of major neurocognitive disorder/dementia or intellectual disability, impairing their capacity to provide consent or reliably fill in the questionnaires. Two medical students were trained by the principal investigator (RK) for a standardized administration of the survey and data collection from patients during their dialysis session. Although many instruments are intended to be self-filled, we opted to administer the entire survey by the trained researchers to increase participation rate and minimize missing data.

### Variables and instruments

- Sociodemographic variables included age, gender, marital status, educational level and socioeconomic status, which was assessed using the household crowding index (number of residents living in the same house divided by the number of rooms).
- Medical comorbidities of participants were also explored, including the duration of dialysis, smoking, alcohol and substance use, presence of comorbid diseases such as cancer, cardiovascular diseases, diabetes, cardiac, hepatic or respiratory failure, rheumatoid diseases, history of strokes, hypothyroidism, Parkinson’s disease, history of COVID-19 infection and/or hospitalization, nutritional status using the Global Leadership Initiative on Malnutrition (GLIM) criteria, the body mass index (BMI), functional assessment/ mobility using the Katz activities of daily living-(ADLs) assessment, frailty using the 5-item “FRAIL” screening tool. Sarcopenia was assessed through the handgrip strength (HGS) measured before the hemodialysis session, using a calibrated arm dynamometer [13]. Sarcopenia was determined using the 2019 revised criteria of the European Working Group on Sarcopenia in Older People (EWGSOP2) by which HGS lower than 27 kg for men and 16 kg for women are indicative of sarcopenia [14].
- Depression and anxiety were assessed using the HADS, a commonly used instrument in hemodialysis patients that is comparable to the Beck Depression Inventory [15, 16]. The Arabic HADS was found to be reliable and valid in Saudi Arabia and in the United Arab Emirates with a Cronbach alpha of 0.73 for HADS-A (anxiety) and 0.77 for HADS-D (depression). In dialysis patients, cut off scores of ≥7 for HADS-D and ≥6 for HADS- A were associated with preferable balance of sensitivity and specificity in end stage kidney disease patients [16]. Those cut-offs were thus used in this study.
- Suicidality was assessed using direct questions regarding wishes/thoughts, plans and attempts.
- PTSD was assessed using the PTSD checklist for DSM-5 (PCL-5), which is a 20-item screener based on the Diagnostic and Statistical Manual (DMS)-5 criteria for PTSD. The cut-off of 23 was used to determine PTSD, based on a previous study that validated the questionnaire in a population of Arabs/Iraqi who were displaced due to the war. This cut-off has been associated with optimal balance of sensitivity and specificity (area under the curve = 0.82, *p* < 0.001; sensitivity = 0.82, specificity = 0.70) [17].
- Cognitive impairment was assessed using the Mini-cog (3 word-recall and a clock drawing test), which is a short screening tool with sensitivity ranges between 80-99%, and specificity ranges between 90-93% [18, 19].
- Other psychosocial variables explored included past psychiatric history, exposure to the Beirut blast, and exposure to lifetime traumatic events (using the life-events checklist per DMS-5; LEC-5). Religiosity was assessed using the Duke University Religion Index (DUREL). It is a 5-item measure of religious involvement and was developed for use in large cross-sectional and longitudinal observational studies. It measures three dimensions of religiosity identified by the National institute of ageing: organizational religious activity (ORA) such as attending church or mosque, non-organizational religious activity (NORA) such as private prayers, and intrinsic religiosity (or subjective religiosity) such as experiencing the divine in daily life. Each dimension is rated on a scale from 1 to 6, going from the least to the most important religious involvement [20]. Scores above 3 on each of the three subscales reflect significant/important religiosity. Social and emotional loneliness were assessed using the 6-item De Jong Gierveld Scale. The three questions assessing emotional loneliness (when someone misses an “intimate relationship”) are negatively worded, and scores were adjusted as follows: the neutral and positive answers are scored as “1”. Therefore, on questions 1-4 and 6 score No=0, the rest=1. The three other questions assessing for social loneliness are positively worded, so the neutral and negative answers are scored as “1”. Therefore, on questions 2-3 and 5, score Yes=0, the rest of the answers= 1. The total score ranges between 0 and 6, from the least to the most lonely [21].
  Health-related Quality of life was assessed using the Short Form 36 (SF-36) health survey, a valid instrument in end-stage kidney disease patients [22]. It contains 36 questions that measures 8 domains: physical functioning, role limitations due to physical problems, bodily pain, general health, vitality, social functioning, role limitations due to emotional problems, and mental health. The instrument assesses two distinct concepts, physical and mental. Although extensively used in the literature, the SF-36 total or global score is not recommended by the developers of SF-36 [23]. The correct calculation of SF-36 specific sub scores requires the use of special algorithms (combining the eight previous domains), which are currently held by a private company. Thus, we resorted to an estimation of the physical and emotional components by applying the average of domains related to physical and emotional components. 4 of the SF-36 subscales were combined to generate a physical health component-PHC (physical functioning, role limitations due to physical problems, bodily pain, and general health perceptions). The other subscales (vitality, social functioning, role limitations due to emotional problems, and mental health) were combined to generate mental health component-MHC. Earlier analyses of the SF-36 tool support the use and validity of these two components [24–26]. Scores range between 0 (poor) to 100 (good).

### Consent/ethics

The study was approved by the Institutional Review Board (IRB) of SGHUMC: IRB-REC/O/007-21/4620. Written informed consent was obtained from all patients or their legal representatives (for illiterate subjects) prior to participation.

### Statistical analysis

The data collected was computed and analyzed using the SPSS software platform, version 21. A Descriptive analysis using numbers and percentages for categorical variables and means with standard deviation for continuous variables was conducted. Prevalence rates of depression, anxiety, suicidality, and PTSD, as well as other medical and psychosocial measures were generated. Our analysis was merely exploratory due to the limited sample size.

## Results

### Sample size

The dialysis unit at SGHUMC provided the investigators with an updated list of 61 adults who were undergoing hemodialysis at the hospital at the time of the study. Eight patients were excluded because of a diagnosis of major neurocognitive disorder or intellectual disability (according to their medical records, and after discussion with their nephrologist). Two patients passed away at the time of the study, before data collection. Nine patients declined to participate. The rate of participation was thus 82.3% (42/51 patients).

### Sociodemographic characteristics

A total of 42 patients were included in the assessment. The mean age of participants was 66.1 years with a standard deviation (SD) of 11.3. 92.9% of participants were aged 50 years or older. Females constituted 45.2% of the sample. Sixty-nine percent were still married, and 83.3% had children; mean (SD) 2.4 (1.7) children. Thirty-one percent of participants had a high school level of education or above. Sixty-six percent of participants belonged to a middle socioeconomic status (SES) and 9.5% to a high SES. Table 1 details the sociodemographic characteristics of our population.

**Table 1:** Sociodemographic characteristics of our population (*N*=42)

Variables	Percentages (count)^a^
**Age**^a^	66.1 (11.2) years
**Gender**	
Female	45.2% (19)
Male	54.8% (23)
**Marital Status**	
Single	19% (8)
Married	69% (29)
Divorced	7.1% (3)
Widowed	4.8% (3)
**Educational Level**	
Middle School Level	52.4% (22)
High School Graduate	14.3% (6)
Technical School	11.9% (5)
University Degree	16.7% (7)
Illiterate	4.8% (2)
**Employment**	
Never employed	33,3% (14)
Previously employed	54.7% (23)
Currently employed	11.9% (5)
**Socioeconomic Status (SES)**	
Low (CRO> 2)	23.8% (10)
Medium (CRO 1-2)	66.7% (28)
High (CRO <1)	9.5% (4)

^a^Mean and standard deviation (SD) were used for continuous variables whereas percentages and absolute numbers were shown for categorical variables

### Medical evaluation

**Fig. 1:**
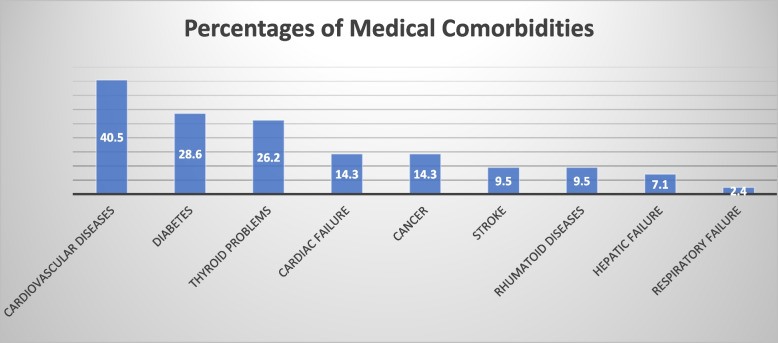
Prevalence of Medical Comorbidities (%), by descending order (*N*=42)

At the time when the study was conducted, patients had been on dialysis for 6.12 years on average with a SD of 7.22 years. Among participants, 59.5% were smokers and 40.5% were alcohol users while 2.4% reported alcohol misuse. In addition, 2.4% reported active drug use. 40.5 % of patients had comorbid cardiovascular diseases, and 26.2 % had thyroid diseases. Figure 1 represents the prevalence rates of all comorbid medical illnesses in our population. 35.7% of the sample had 2 medical comorbidities, while around 17% of the sample had 3 or more comorbidities. The mean BMI of our population was 27.46 (SD: 6.44).

Around one third of our sample (28.5%) were found to be frail, according to the FRAIL screening questionnaire while 54.8% met criteria for pre-frailty. Moderate to severe functional impairment, according to the Katz scale was found in 16.7% of the sample. Based on the GLIM criteria, 71.4% were found to be moderately to severely malnourished.

The mean HGS was found to be 23.9 (SD:10.3) and 15.6 (SD:7.7) in men and women respectively. It was found that 59.5% of our sample suffered from sarcopenia according to the EWGSOP2 grip strength criteria (considering gender difference). At the time of the assessment, 16.7% (7 out of 42 patients) had already contracted COVID-19 infection, of whom 7.1% (3 patients) were hospitalized.

### Anxiety, depression, PTSD, and cognitive assessments

**Fig. 2:**
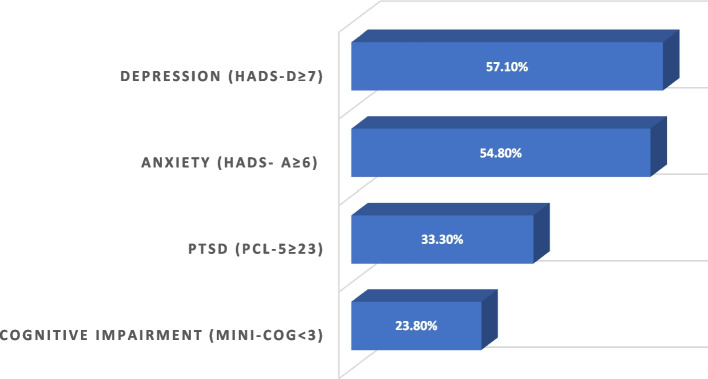
Screening for Psychiatric Disorders in Lebanese Hemodialysis Patients (*N*=42). Abbreviations: HADS-D: Hospital Anxiety and Depression Scale- Depressive Scale; HADS-A: Hospital Anxiety and Depression Scale- Anxiety Scale; PTSD: Post-Traumatic Stress Disorder; PCL-5: PTSD Checklist for Diagnostic and Statistical Manual- 5^th^ version- DSM-5

Anxiety and depression rates were found to be significantly high, reaching 54.8% and 57.1% using cut-offs of six and seven respectively on the HADS. 26.2% had passive death wishes and 7.1% had suicide plans, however no one had attempted suicide. 33.3% of our population screened positively for PTSD, using the PCL-5. Around a quarter of the sample (23.8%) were found cognitively impaired as shown by the Mini-Cog (<3), despite no previously established dementia diagnosis. The data is presented in Fig. 2. Only 12% had visited a mental health professional.

### Trauma exposure

Four out of 42 patients (9.5%) were at the hospital on the day of the Beirut explosion, of whom three were injured. Eight patients (19%) had close relatives who either passed or were severely injured. More than half of the sample (54.7%) had their home either partially or entirely destroyed by the explosion. Around a quarter of the population (23.8%) saw dead or mutilated bodies on the day of the explosion. The inventory of lifetime traumatic events showed that the median number of traumatic events was six; mean 6.14 (SD: 3).

### Other psychosocial measures

30.9% had ORA scores> 3; 83.3% had NORA scores >3, and 88% had significant subjective meaning of religion in their heart.

59.5% of the population reported feeling moderate to severe loneliness (total score ≥3).

26.2% of participants reported extreme scores on measures of social loneliness, contrasting with only 2.4% reporting extreme emotional loneliness, while 52.4% and 54.8% denying social and emotional loneliness, respectively.

On a scale from 0 (poor) to 100 (good), mean values for physical and mental health functioning/quality of life were 55.1 (SD=23.8) and 67.6 (SD=21), respectively.

## Discussion

Depression and anxiety are the most common mental health disorders in patients on dialysis, and are associated with poor quality of life, and increased morbidity and mortality. Studies from around the world have found a wide disparity between prevalence rates, depending on sample size, instruments used, and ethnic differences between populations [27].

Our study showed relatively higher rates of anxiety and depression (55 and 57% respectively), compared to previous measures in patients undergoing hemodialysis in other Lebanese hospitals, prior to the blast (40-50%) using the same screening instrument, the HADS [11, 12]. These rates are among the highest in the Arab region: in Saudi patients on hemodialysis, rates ranged between 20-21% [28, 29] for anxiety, and 23-43% for depression [28–30]. In Morocco, anxiety and depression were found in 25 and 34% of patients, respectively, using the Mini-International Neuropsychiatric Interview [31]. In Kuweit, depression and anxiety were found in 21% of participants using the HADS [32]. Suicidal rates were also the highest in our study: suicidal thoughts were found in 26% of the participants compared to 16.5% of the Moroccan patients. 7% of our sample planned suicide compared to 1.9% of the Moroccan patients [31].

Higher rates in our sample can be evidently attributable to the declining socioeconomic situation, stress related to the COVID-19 pandemic, as well as the traumatic August 4 explosion, and echo findings from neighboring areas with similar unstable political and socio-economic conditions. One study from West Bank, an occupied Palestinian territory, showed higher rates of depression, reaching 73%. In this sample, correlates of depression involved older age, low socioeconomic status, living situation in rural areas/camps, and multiple comorbidities [33]. In Iraq, depression rates reached 80% using the DSM-IV criteria, as well as the Beck Depression inventory [34]. Explanations to higher rates in these studies include the use of a different instrument the Beck Depression Inventory (BDI) scale, and the repetitive exposure to traumatic war-zone conflicts or events. Similarly, in another study comparing Syrian refugees to Turkish participants undergoing hemodialysis, rates of depressive symptoms using the BDI were significantly higher in the group of Syrian refugees compared to Turkish (72.7% compared to 42.7%) [35], postulating the possible impact of the Syrian civil war and PTSD on the emergence of depressive symptoms in these populations.

Our study is the first to explore PTSD prevalence rates in patients on hemodialysis in Lebanon and the Arab countries. Indeed, trauma and PTSD are under investigated in hemodialysis patients. More than a third (33%) of our population screened positively for PTSD. This number is higher than global prevalence figures in hemodialysis patients, following a natural disaster. For instance, in the year following hurricane Katrina in New Orleans, United States, 23.8% of hemodialysis patients reported symptoms consistent with PTSD using the PCL-17/DSM-IV [36]. This difference could be attributed to the cumulative number of traumatic experiences that has been affecting the Lebanese population since the 1975 war [37]. Our study demonstrated that the mean number of lifetime traumatic events was 6 in this sample of Lebanese hemodialysis patients. Cumulative trauma load was found to be associated with a higher likelihood of developing PTSD and a greater severity or complexity of the disorder [38]. In one American database analysis study from the general population, participants who had only one type of trauma exposure had a 0% likelihood of current PTSD, whereas those with 6 or more other trauma types had a 12% likelihood of developing this disorder [39].

Our findings on PTSD among hemodialysis patients are comparable to data from a Turkish cross-sectional study that showed 33.3% of Syrian refugees undergoing hemodialysis suffered from PTSD (using the PCL-5, with a cut-off of ≥31). This rate was significantly higher than PTSD rates in Turkish native patients undergoing hemodialysis [40]. This is understandable given that the magnitude of the Beirut blast may be comparable to the atrocities of war and displacement suffered by the Syrians.

Cognitive impairment is common and undiagnosed in patients on hemodialysis, due to their older age, and increased cardiovascular comorbidities, notably the presence of strokes [41]. It can also be exacerbated by comorbid psychiatric illness, including depression, anxiety, and PTSD such as in our study. In our sample patients with no prior dementia diagnosis, one third of participants were shown to be cognitively impaired. The prevalence of cognitive impairment among patients on hemodialysis, assessed using neuropsychological tests, varies from 16 to 38% [42], whereas this prevalence ranged from 6.6 to 51%, using screening tests. The most used ones in the literature were the Mini-Mental State Examination (MMSE), the Montreal Cognitive Assessment (MoCA) and the Modified Mini-Mental State (3MS). Our study is the first to use the Mini-Cog [43], which is a short and practical tool considered as a quick “vital signs” measure for cognition [18]. It is of utmost importance to shed light on the importance of assessing for cognitive function before initiation of dialysis and periodically, even by using a brief cognitive scale, especially that cognitive decline was shown to be an independent risk factor for all-cause mortality in patients on hemodialysis [44].

On the other hand, an alarming percentage (more than 80%) of our sample was found to be pre-frail or frail. There is a bidirectional relationship between frailty and depression in patients on hemodialysis. In a cross-sectional study involving 80 patients on hemodialysis, patients suffering from depression were 9.8 times more likely to be frail than were patients without depression (odds ratio, OR = 9.80; 95% confidence interval, CI, 1.93-49.79) [45]. In addition, frailty was found to be an independent risk factor for developing depression in this population: there is thus a linear relation between a greater frailty severity and the probability of depression [46]. Frailty and sarcopenia were also found to be associated with increased risks of falls, hospitalization, institutionalization, and mortality [47]. Nutritional status has been associated as well with both depression and anxiety in hemodialysis patients. In a sample of 55 adult patients on hemodialysis, depression and anxiety prevalence rates as measured by the HADS were found to be significantly higher in those with poor nutritional status as assessed via the mini-nutritional assessment (MNA). In addition, those with depression and anxiety had a significantly higher risk of developing malnutrition [48]. Two-thirds of our sample had moderate to severe malnutrition according to the GLIM criteria and around 60% suffered from sarcopenia according to HGS measures. Thus, frailty, sarcopenia and malnutrition are essential components that need urgent intervention in our studied population.

When compared to data from the literature, Lebanese patients on hemodialysis reported acceptable to good quality of life on both physical and mental health components: mean scores on these two variables using the SF-36 were shown to be 55.1 and 67.6 compared to 49.4 and 38.7, respectively in a Saudi study using the same instrument [49]. Quality of life is an individual’s perception of their position in life with respect to their culture, value system, and relationship to life goals, expectations, and standards. Determinants of quality of life in this population include socioeconomic status, and number of comorbidities. Nutritional status has not been found to be correlated with quality of life in these patients [50]. The subjective report of good quality of life of our participants can be linked to the fact that 76.2% of participants had middle to high socioeconomic status, and half of our sample had a low number (0-1) of medical comorbidities, despite an increased prevalence of undiagnosed mental illness. Furthermore, moderate to severe impairment in activities of daily living assessed using the Katz was found in less than 20% of the sample, which could have impacted their vision of their quality of life. Those are reassuring prognostic factors as both quality of life and impairment in daily functioning have been shown to be independent risk factors for increased mortality in patients on hemodialysis [51].

To date, studies that explored the association between religiosity/spirituality and quality of life as well as risk of developing depression have mixed results, notably in patients with chronic illnesses such as end-stage kidney diseases. The results may have been modulated by the measure of spirituality used in studied populations, whether it is practicing of the religious rituals socially or privately or using faith as a coping mechanism with the burden of illness [52]. The current study aimed to assess religiosity through three different angles: organizational, non-organizational and subjective. Interestingly, only a third of the sample attended religious activities, compared to more than 80% who prayed alone, or gave a subjective meaning to religion in their daily life. This data was however collected amid the COVID-19 pandemic, in the setting of firm restrictions imposed on religious practices in worship places. In a Saudi study, 158 patients on hemodialysis answered the 13-item Muslim Religious Index, which includes two subscales, namely religious practices scale (10 items) and the intrinsic religious beliefs scale (3 items). Patients aged over 50 years showed significantly higher involvement in religious practice and intrinsic religiosity compared to their younger counterparts [53]. This is relevant as religion is viewed as the most important marker of communal identity in the Middle East, shaping individuals’ patterns of coping and adaptation to stressors, including war traumas. Inconsistent associations between religiosity and mental health disorders have been reported in the literature, depending on multiple factors including but not limited to affiliation, sect, gender, age and ethnicity [54]. Religiosity in both its organizational and nonorganizational forms has been shown to be a protective factor against depression and to help individuals recover from depression [55]. This protective effect has been particularly evident in older women reporting religious affiliation or attending religious services more than once weekly [56]. In a study of Veterans in PTSD residential treatment, higher levels of extrinsic-social religious motivation were associated with lower severity of PTSD, while a more negative concept of God was associated with higher severity of PTSD [57]. In a cross-sectional study of American veterans and active-duty military, organizational and cognitive/intrinsic religiosity were shown to be inversely correlated to negative cognitions/emotions (criterion D symptoms of PTSD) only, with no significant impact on other PTSD criteria [58]. Data on the impact of religiosity on PTSD occurrence and severity in Middle Eastern populations is lacking.

Our study is unique in terms of the vulnerable and under-investigated population studied and the multiple psychosocial variables that were assessed, notably history of trauma and emergence of PTSD symptoms, in the year following a major collective traumatic event. Although the questionnaires are usually self-filled, they were administered under the supervision of trained investigators to minimize missing data and misunderstanding of questions. Limitations include the use of screening instruments, instead of diagnostic tools or interviews. The screening tools used have not been validated in the Lebanese population, and thus normative data from neighboring Arab countries were used (i.e. the cut-off value of the PCL-5 was used from the Iraqi study by Ibrahim and colleagues [17]). In addition, the research was performed on a small sample size (only 42 participants), which didn’t allow to perform bivariate or multivariate analyses and study correlates of developing depression, anxiety, and PTSD. The results presented are thus descriptive in nature and would not infer causality or correlations between the different variables. Data was collected from a single center and results are not necessarily representative of the Lebanese population on hemodialysis.

## Conclusion

In an urban dialysis unit, affected by a major explosion, a declining socioeconomic situation and the COVID-19 pandemic, depression, anxiety, PTSD, and cognitive impairment were found to be highly prevalent, urging the need for a prompt psychiatric evaluation in similar settings and early intervention strategies aiming at improving the physical and mental well-being in this vulnerable population. This project sheds light on this vulnerable population that is understudied in the region and the world. Early diagnosis and treatment of mental illness will not only improve quality of life of patients on hemodialysis but will decrease mortality rates. Larger multicentered studies are however needed to have a more representative perspective on mental illness prevalence and its determinants in patients undergoing hemodialysis in Lebanon and the region. Validation studies and determination of normative data for assessment tools for PTSD such as the PCL-5 or shorter screening tools are much needed, notably in vulnerable populations such as older adults and those on hemodialysis, to perform more solid research in this field in Lebanon and the region.

## Data Availability

The dataset used and analyzed during the current study are available from the corresponding author on reasonable request.

## Abbreviations

SGHUMC: Saint Georges Hospital University Medical Center
PTSD: Post-traumatic stress disorder
MERS: Middle East Respiratory Syndrome
HADS: Hospital Anxiety and Depression Scale
GLIM: Global Leadership Initiative on Malnutrition
BMI: Body mass index
ADL: Activities of daily living
HGS: Handgrip strength
EWGSOP2: European Working Group on Sarcopenia in Older People
DSM: Diagnostic and Statistical Manual
PCL-5: Post-traumatic Stress Disorder Checklist for DSM-5
LEC-5: Life Events Checklist for DSM-5
DUREL: Duke University Religion Index
ORA: Organizational religious activity
NORA: Non-organizational religious activity
SF-36: The Short Form 36 health survey
PHC: Physical health component
MHC: Mental health component
IRB: Institutional Review Board
SD: Standard deviation
SES: Socioeconomic status
BDI: Beck Depression Inventory
MNA: Mini-nutritional assessment
CRO: Crowding Index
